# The Diet Quality of Athletes as Measured by Diet Quality Indices: A Scoping Review

**DOI:** 10.3390/nu17010089

**Published:** 2024-12-29

**Authors:** Stella Dion, Gabriel Walker, Kelly Lambert, Anita Stefoska-Needham, Joel C. Craddock

**Affiliations:** School of Medical, Indigenous and Health Sciences, Faculty of Science Medicine and Health, University of Wollongong, Wollongong, NSW 2500, Australia

**Keywords:** athlete, diet quality, diet quality index, sports, nutrition, scoping review

## Abstract

Background/Objectives: The goals of sports nutrition are to support athletic performance. However, the diet quality of athletes remains poorly understood. This scoping review aims to synthesise the existing body of literature, evaluating the diet quality of adult athletes. Methods: The scoping review was conducted in accordance with guidance from the Joanna Briggs Institute. Database searches were conducted in Medline, Scopus, SPORTDiscus, Web of Science, and EMBASE. Eligible studies were observational, utilised a validated dietary quality assessment tool (diet quality index), and assessed athletes aged 18 to 65 years. Screening was performed independently by two researchers, with any conflicts resolved by a third researcher. Results: The search yielded 1142 articles, of which 18 met the inclusion criteria. Basketball athletes and gymnasts were the most frequently examined groups. Studies were dominated by one gender (*n* = 7 all males studies, *n* = 4 only female). Eleven diet quality tools were used, including variations of the Healthy Eating Index (*n* = 7). Overall diet quality was rated as poor (*n* = 3 studies), needs improvement (*n* = 12), or adequate (*n* = 3). Food groups where intake was suboptimal included wholegrains (*n* = 8), fruit (*n* = 5), and dairy intake (*n* = 3). The intake of protein-rich foods was adequate in *n* = 9 studies, whilst fat intake was excessive in 4 studies. Conclusions: These findings suggested that the diet quality of athletes is suboptimal and needs improvement in the areas of wholegrain, fruit, and dairy/alternatives intake. The inadequate intake of these food groups and the consumption of excessive fat intake may increase the long-term risk of developing non-communicable diseases and impair short-term performance and recovery. Further exploration is warranted to develop targeted nutrition education and interventions that address these inadequacies.

## 1. Introduction

Optimising an athlete’s nutritional intake is a well-researched and accepted approach for improving performance and recovery [1,2]. Various dietary approaches can contribute to improved athletic performance [2]. For instance, in endurance sports, dietary recommendations focus on carbohydrate consumption prior to, during, and following the conclusion of training and competition due to their ergogenic properties and role in recovery [3,4]. Performance-focused nutrition in strength- or power-based sports involves the manipulation of protein intake, particularly post-training, to promote muscle protein synthesis, strength, and hypertrophy [5]. Such dietary optimisation is influenced by the athlete’s specific sporting modality (i.e., strength-based vs. endurance-based), individual goals, and personalisation factors, such as individual food preferences, cultural dietary preferences, dietary intolerances and allergies, and gastrointestinal tolerance around training [6].

Athletes often use sports nutrition products or supplements and ergogenic aids to achieve specific optimal performance and recovery outcomes [7]. A recent review demonstrated that the prevalence of these products among athletes ranged from 11% to 100%, depending on a number of variables, including sport modality, sex, and level of competition [8], with sports supplement usage being particularly pronounced in endurance-based sports. A study conducted by Heikura et al. (2018) reported that of 104 elite male and female middle- and long-distance competitive runners, 90% relied solely on sports nutrition products in the form of drinks, gels, chews, and bars to meet their carbohydrate needs during training and events [9]. Similarly, to meet increased needs, reliance on protein supplementation in athletes was prominent across a range of sports and dietary patterns [10,11,12]. Whilst this approach to fuelling may be ideal for performance, consistent reliance on sports nutrition products and supplements may compromise the quantity and variety of whole foods consumed, potentially leading to nutrient deficits and reduced diet quality.

To foster health and wellbeing and reduce the risk of non-communicable chronic diseases, it is essential to consume a nutritionally dense diet incorporating a variety of foods from the core food groups outlined in global dietary guidelines [13]. Additionally, eating food according to a high-quality dietary pattern may contribute to the alleviation of exercise-induced immunosuppression and may also reduce the risk of injury [14,15,16]. Evidence suggests that regularly adhering to a high-quality diet may allow athletes to train more consistently, which, in turn, could positively impact their performance [17,18]. For example, dietary patterns rich in flavonoids and abundant in fruits and vegetables are associated with a reduced prevalence of chronic respiratory diseases, potentially mediated by systemic inflammation [19]. Therefore, diet quality in athlete populations is important for both health and well-being, as well as performance outcomes. Despite this link, diet quality remains infrequently considered in studies of athlete populations.

Diet quality indices (DQIs) are routinely used to assess the quality of an individual’s diet. DQIs are research instruments that are underpinned by current nutritional knowledge and typically grade dietary intake based on a criterion of nutrients, food groups, or recommendations in dietary guidelines [20]. DQIs represent standards of general “healthiness” of an individual’s dietary pattern and food intake [21]. DQIs, like the Healthy Eating Index [22], track dietary quality against dietary guidelines, while others, like the Mediterranean diet (MedDiet) food checklist, measure adherence to specific dietary patterns [23]. No synthesis of research evidence was conducted on the diet quality of athletes, despite its potential significance for elite athletes’ health and performance. Thus, the aim of this scoping review was to synthesise the existing body of evidence, evaluating the diet quality of adult athletes, as measured by validated DQIs.

## 2. Materials and Methods

### 2.1. Study Overview

This scoping review was conducted as per the Joanna Briggs Institute manual for scoping reviews, as well as the Preferred Reporting Items for Systematic Reviews and Meta-Analyses extension for Scoping Reviews (PRISMA-ScR) [24,25]. The research question was developed using the Population Concept Context (PCC) format and was registered with the “open science framework” (Registration DOI https://doi.org/10.17605/OSF.IO/XDUW7 accessed on 26 December 2024) in accordance with the PRISMA-ScR extension [24,25].

### 2.2. Search Strategy

To inform the search strategy, a pilot search of the literature available on the Medline and Scopus databases was performed using the following search string (“athlete” OR “sport”) AND (“diet quality” OR “diet index”). The search retrieved 304 results, and the titles and abstracts of approximately half of these were screened to identify keywords and MeSH terms relevant to the topic. Sentinel articles were also retrieved to help refine and test the search strings. The “index terms” section of articles were also screened for additional relevant terms. A revised search including all identified terms found during the pilot search was then performed across the Medline, Scopus, SPORTDiscus, Web of Science and Embase databases using the following search terms (“athlet*” OR “sport*”) AND (“diet quality” OR “diet index” OR “healthy eating index” OR “HEI” OR “DQI” OR “diet score” OR “healthy eating score” OR “HEIFA-2013”). The inclusion of these specific terms was based on their frequent use in existing studies, as well as the findings from the initial pilot search, aiming to capture relevant dietary assessment tools commonly applied in athletic populations. This search was limited to the title, abstract, and keywords. No other limitations were applied.

### 2.3. Inclusion Criteria

Eligible studies were required to be peer-reviewed observational in design and to use a validated DQI to evaluate the dietary quality of athletes between the ages of 18 and 65. For the purpose of this review, an athlete was defined as someone who trains in a manner specific to their sport, at least three times per week, and is engaged in local-level representation, at a minimum, as outlined by McKay et al. (2022) [26].

### 2.4. Exclusion Criteria

Studies of athletes aged >65 years were excluded as the nutritional requirements and physiology of people above 65 years differ from younger adults [27,28]. Studies were also excluded if participants did not fit the definition of an athlete or included participants that were pregnant or suffered from one or more chronic disease (e.g., type 2 diabetes mellitus), aligning with McKay et al.’s (2022) framework [26]. Studies were also excluded if they were not available in English, were conference abstracts, included participants under the age of 18 years, failed to report results in a stratified manner of 18–65 years, or did not use a validated DQI to evaluate diet quality. While peer-reviewed articles were sought to form the main body of evidence for this review, non-peer-reviewed academic articles such as student theses, were also synthesised (Supplementary Material—Table S1) to provide a holistic view of all research in the athlete and diet quality space.

### 2.5. Study Selection

All search results were exported to Covidence [29], where duplicates were removed. The titles and abstracts of the articles were then screened against eligibility criteria. Full-text versions of the remaining articles were obtained and screened. Screening was performed in duplicate by two researchers. The lead researcher helped to resolve any arising conflicts. Following full-text screening, the reference sections of included articles were hand searched to identify additional relevant studies meeting the inclusion criteria.

### 2.6. Data Extraction

Relevant data were extracted from included articles, including the following: primary author(s), publication year, country in which the study was conducted, participant/athlete characteristics, sample size, dietary assessment tool implemented, DQI used, and any relevant findings. Means and standard deviations, or medians and interquartile ranges, were used to summarise DQI scores.

## 3. Results

**Figure 1:**
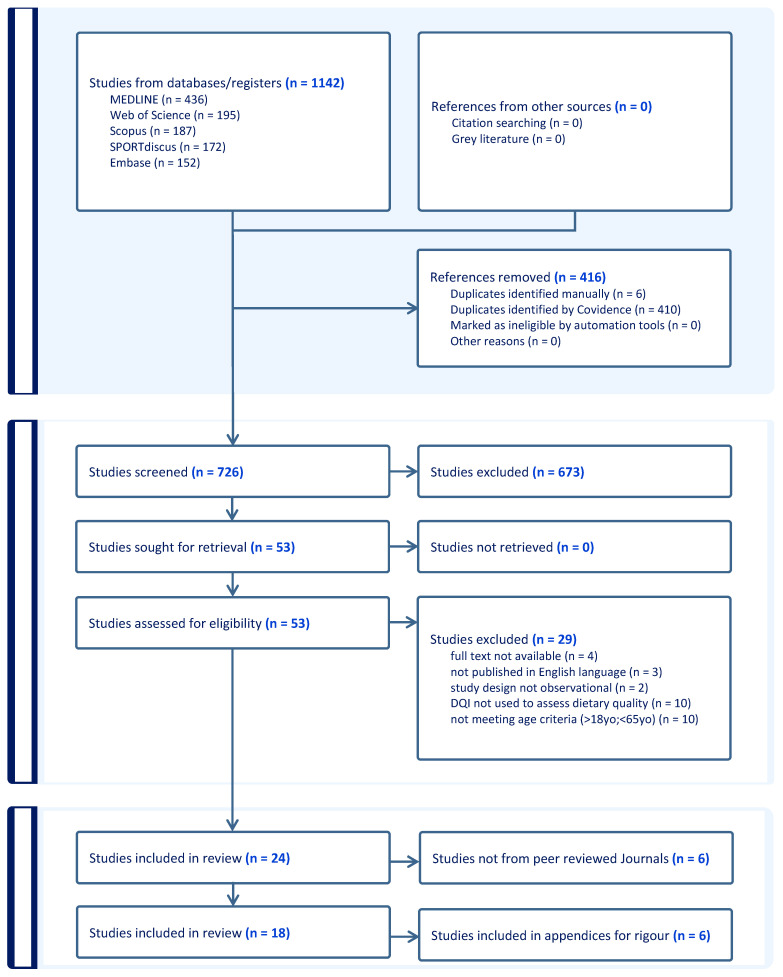
Flow chart of study screening.

The search retrieved 1142 articles across the five databases (Medline = 436, Scopus = 187, SPORTDiscus = 172, Web of Science = 195, and Embase = 152; Figure 1). Following the removal of duplicates, 726 articles underwent title and abstract screening. Twenty-four articles met the inclusion criteria and were reviewed. Eighteen of these studies were peer reviewed and were subsequently included in this review, whilst six were non-peer reviewed and form Supplementary Material—Table S1. No additional relevant articles were found through the hand searching of reference lists.

### 3.1. Study Characteristics

All included articles were published from 2010 to December 2023 (Table 1). Across the studies included in this review, athletes were sampled from 25 different sports. Basketball athletes and gymnasts were most frequently examined featuring in 5 of the 18 studies retrieved [30,31,32,33,34,35,36,37]. Other sports from which athletes were frequently sampled included swimming (*n* = 3) [31,35,36] and soccer (*n* = 4) [30,31,35,38]. Most studies (*n* = 6) retrieved examined dietary quality in college-level athletes [31,35,36,37,38,39], and *n* = 4 studies examined elite/Olympic-level professional athletes [32,33,40,41]. Eight studies were conducted in the USA [31,35,36,37,38,39,42,43], six in Europe [30,33,44,45,46,47], two in South America [40,41], one in Australia [48], and one that was conducted on a multinational scale [32]. Thirteen studies were cross-sectional in design [30,31,32,33,35,39,40,42,43,44,45,46,47], and five were longitudinal [36,37,38,41,48]. Five studies had less than 50 participants [31,33,37,40,48]; four had between 50 and 100 [35,38,44,47]; eight had between 100 and 500 [30,36,39,41,42,43,45,46]; and one study had more than 500 participants [32]. The age of participants ranged from 18 to 65. Seven studies included only male participants [30,33,38,44,46,47,48], and four included only female participants [31,37,39,42].

### 3.2. Dietary Collection Methods

In total, 12 dietary collection methods were used, all of which were adaptations of either 24 h recalls, Food Frequency Questionnaires (FFQs), or food records that varied in design, collection method, and/or duration. Specifically, five articles used validated derivatives of FFQ variations [30,36,45,46,47]. More specifically, three of five studies used the KomPan Dietary Habits and Nutrition Beliefs Questionnaire [30,45,47], and the two remaining studies implemented the VioScreen, a web-based, graphical FFQ [36], or the Australian Recommended Food Score (ARFS) [46], respectively. Four studies utilised food records ranging from three to seven days of intake [37,40,44,48], and six studies used 24 h recalls [31,33,35,38,41,42] to assess dietary intake. In relation to the administration of the dietary data collection, five of the eighteen studies were interview facilitated [31,33,41,47], and nine were completed independently by the participant(s) [30,35,36,37,42,43,44,45,48]. Of the unsupervised dietary collection methods, four groups received some form of guidance or training [36,37,44,48], and four did not [35,42,45,46].

### 3.3. Diet Quality Indices (DQI’s)

In total, eleven DQIs were used, seven of which were variations of the Healthy Eating Index (HEI). The HEI-2015 was the most frequently used (six studies) [31,35,36,37,38,42], followed by the Pro-Healthy Diet Index (pHDI-10), which was used in three studies [30,45,47]. Eleven of the studies used DQIs informed by the various iterations of the Dietary Guidelines for Americans (DGA) [31,32,33,35,36,37,38,39,42,43,44]; two utilised the Australian Dietary Guidelines [46,48], and two used tools adapted from the Brazilian Dietary Guidelines [40,41]. Three used DQIs that were not informed by dietary guidelines but rather food groups or food components which are thought to positively (i.e., legume-based foods) or negatively (e.g., white bread) influence health [30,45,47].

### 3.4. Diet Quality Scores

Based on the scoring systems of the DQIs implemented, three studies found that athletes had poor quality diets [30,38,47], nine studies found diet quality was suboptimal and needed improvement [31,35,36,37,40,41,42,44,46], and one study found that athletes reported adequate diet quality [33]. The four remaining studies utilised DQIs or proxy tools/surveys in place of DQIs without scoring stratification; in these cases, higher scores indicated better diet quality [32,39,43,48]. Table 2 provides a summary of the diet quality exhibited in the athlete groups identified in this review. Among studies employing a dietary quality measure with a continuous outcome, Turner-McGrievy et al. found that elite and sub-elite participants achieved, on average, 65% and 64% of the maximum score available, respectively, indicating suitable adherence to healthy eating principles [43]. Seven of the nine studies reporting suboptimal dietary quality had scores closer to the lower end of the scale, indicating diets closer to poor quality than adequacy [31,35,37,40,41,44,46]. The food groups most commonly found to be inadequate included wholegrains [30,31,35,36,40,42,46,47], fruits [30,31,34,40,46], and dairy products [30,40,47]. Protein foods were most commonly consumed in adequate quantities [33,35,36,37,38,40,42,44,46], whilst fats were most commonly consumed in excess [34,38,40,42]. There were no apparent differences in diet quality between athlete types or sporting modalities.

## 4. Discussion

Overall, the findings indicated that there is a limited body of research describing the dietary quality of athletes. Moreover, numerous inconsistencies were identified among studies regarding methodologies for assessing diet quality in athletes. Some studies utilised formal DQIs, while others relied on proxy tools for indirect estimation of diet quality (e.g., Rapid Eating and Activity Assessment for Patients [REAP] [50]). Additionally, a variety of dietary collection methods were identified, including 24 h recalls, FFQs, and food records, each differing in design, collection approach, and/or duration. These variations make the comparison of diet quality between studies challenging. Nonetheless, the findings of this research indicated that the diet quality of adult athletes was largely suboptimal. Common areas of inadequacy included the suboptimal intake of wholegrains, fruits, and dairy products. Protein rich foods were generally consumed in adequate quantities, whilst fat intake was excessive. These dietary trends may reflect a combination of factors, including athlete behaviour, nutritional education, and accessibility to whole foods. Athletes often prioritise macronutrient intake, particularly protein [51], to support training demands, which may explain the adequate consumption of protein-rich foods. However, the lower intake of wholegrains, fruits, and dairy could be attributed to a lack of awareness or education regarding the importance of these food groups for overall health, performance, and recovery. Additionally, the excessive intake of fats may be influenced by the convenience and availability of high-fat processed foods, which are often more accessible than nutrient-dense whole foods, particularly for athletes, potentially with limited time for meal preparation.

Although this review revealed suboptimal diet quality among athletes, their scores still exceeded those of the general public. For example, Tao et al. (2022) evaluated dietary data from 19,192 American adults in the 2018 American National Health and Nutrition Examination survey (NHANES) using the 2015 Healthy Eating Index (HEI-2015). The mean score was 52.65%, suggesting the need for dietary improvements in the general population to better align with the dietary guidelines for Americans [52]. In the present review, we found the majority of studies utilising the HEI-2015 to evaluate the diet quality of American athletes reported higher mean participant scores, ranging from 56.2 to 73.5% [31,36]. This difference may be attributed to athletes’ heightened awareness of nutrition’s role in performance, coupled with greater access to sports nutrition education and resources. These findings underscored important health implications for both groups. For instance, validated dietary quality index tools like the Alternate Healthy Eating Index (AHEI-2010) demonstrated a strong inverse correlation with inflammatory markers [53] and the risk of chronic diseases [54]. Similarly, other DQIs, like the HEI-2015, demonstrated inverse associations with all-cause mortality, type 2 diabetes, cancer incidence and mortality, cardiovascular disease (CVD) incidence, and neurodegenerative diseases [55], suggesting that athletes may face risks of developing these non-communicable diseases.

Low DQI scores typically result from inadequate intake of core food groups and/or excessive intake of nutrients that are detrimental to health [56]. In this review, inadequate intake of wholegrains, fruits and dairy products was most frequently observed, which is concerning as these foods are rich in health-promoting vitamins, minerals, phytochemicals with antioxidant properties, dietary fibre, protein, and calcium [57,58,59]. Additionally, these foods and nutrients were shown to play a role in mitigating pathogenesis, and when consumed regularly in the diet, they may help to prevent the development of chronic diseases. According to a meta-analysis by Ye et al. (2012), individuals consuming three to five serves of wholegrains per day experienced a 21% reduction in type 2 diabetes and a 26% reduction in CVD risk, compared to those who rarely or never ate wholegrains [60]. This may be partially explained by the composition of wholegrains (up to 50% soluble fibre), which may lower LDL cholesterol and improve post-prandial glucose responses when consumed regularly [60,61]. Additionally, fruit intake was observed to favourably impact hypertension, gut health and serum LDL cholesterol levels and was associated with lower risks of T2D, CVD, and some cancers [58,62]. Of concern, the excessive consumption of solid fats like butter, shortening, or lard was reported in a number of studies included in this review [34,38,40,42]. Dietary guidelines typically recommend limiting solid fats due to their association with elevated serum LDL cholesterol and the development of CVD [63,64].

The suboptimal diet quality observed among athletes in this review can be attributed to several factors. Firstly, athletes often prioritise specialised foods like sports drinks, gels, chews, and protein supplements for performance enhancement and optimising body composition, potentially leading to a reduced intake of whole foods [8,9,10,11,12]. The heavy reliance on processed sports nutrition products may also result in potential nutrient deficiencies, as these products often lack essential micronutrients and fibre found in whole foods. Over time, this could increase the risk of long-term health issues, such as cardiovascular disease, poor gut health, or metabolic imbalances [65,66], especially if whole food consumption remains insufficient. Secondly, while elite athletes may have regular access to nutrition professionals, many lower-level competitors and recreational athletes, who were prominent in this scoping review, may not [2]. Limited access to nutrition professionals could explain the low scores observed in this review, as evidence suggests that interactions with nutrition professionals can improve diet quality within athlete populations [36]. Thirdly, sample bias may be a factor. For example, nearly half the studies in this review examined diet quality in collegiate athletes. College students were shown to have sub-optimal fruit and vegetable intake and excessive consumption of discretionary foods, potentially impacting diet quality [67,68,69]. Finally, several studies used DQIs based on dietary guidelines of other countries, possibly not fully representing the culture and cuisine of the resident athlete populations. While DQIs were created for general use in populations, future research could explore the development and application of region-specific DQIs that better reflect local dietary practices, cultural food preferences, and availability of food sources. Additionally, given the differing nutritional requirements of athletes, there is potential to create athlete-specific DQIs to more accurately assess their diet quality and the impact on performance.

This review found that athletes generally consume sufficient quantities of protein-rich foods. However, this finding can be misleading because most DQIs do not differentiate between adequacy and excess. For example, if a DQI awards maximum points for consuming three serves of protein foods, but an individual consumes eight serves, it would still be scored as adequate intake, despite the excess five servings. Although athletes require increased protein intake [70], they far often exceed these requirements [51]. The increased intake of protein-based foods might displace other nutrient-dense options (such as wholegrains), thereby contributing to suboptimal diet quality scores.

With limited studies on diet quality in athletes, the impact of diet on performance in athlete populations may be underestimated. The consumption of nutrient-rich, high-quality, plant-dominant diets were linked to improved blood viscosity, arterial compliance, and vascular flow [71], potentially enhancing tissue oxygenation and performance [71]. Additionally, high-quality diets inherently contain food components with high antioxidant potential that can mitigate exercised-induced inflammation [72], possibly improving recovery, reducing injury risk, and indirectly modulating performance [14,15,16]. Raysmith et al. (2015) highlighted that the inability to train due to injury or illness hampers the performance of elite track and field athletes competing at an international level [17]. Therefore, suboptimal diet quality not only raises the risk of developing chronic diseases in athletes such as type 2 diabetes, cardiovascular disease, and obesity [66] but also affects their performance and recovery.

This synthesis extends our understanding of the diet quality of athletes. Use of rigorous methodology [24] to capture and map the evidence base is a strength and minimises the risk of methodological error [73]. Additionally, the JBI methodology recommends using a minimum of three diverse databases to conduct scoping reviews to decrease the likelihood of overlooking eligible articles, resulting in false conclusions being drawn [74]. Thus, the use of five databases in this review is a strength [24]. This review also identified a lack of studies evaluating diet quality in several areas, including the following: in elite athletes, across a breadth of sports, within many countries (most studies identified in this review were based in the United States), across genders in some sports, and following athletes longitudinally during various training phases over the course of a season or career. On the other hand, this study also has several limitations. Grey literature was not searched; therefore, the findings from this review may not fully represent the evidence base. Additionally, reports of the diet quality of Olympic-level and highly elite athletes may be subject to privacy and confidentiality constraints and not be available in the public domain. Future research could explore these elements, as well as barriers to achieving improved diet quality. Consensus regarding the reporting of diet quality among athlete populations is also recommended to ensure comparability between studies.

## 5. Conclusions

This review highlighted the suboptimal diet quality observed among athletes, which may pose risks to both health and performance. While athletes typically exhibit better diet quality than the general population, it appears many still fall short of nutritional recommendations. Key inadequacies identified included the suboptimal intake of wholegrains, fruits, and dairy or alternative food groups. To address these challenges, targeted nutrition education and culturally tailored dietary interventions could improve athlete diets across diverse populations. Moreover, developing athlete-specific dietary quality indices could offer a more accurate reflection of their diet quality, as traditional DQIs, designed for general populations, may not fully capture the unique dietary needs of athletes.

## Figures and Tables

**Table 1:** Summary of included articles.

Author/s (Year)	Country	Athlete Type	Age	*n*	Dietary Assessment Method	Diet Quality Index Tool	Findings *
Craddock et al. (2023) [48]	Australia	Male endurance athletes > 4 h training > 4 h/week (8 omnivorous, 12 vegan)	18–55	20	7-day food diary	aHEI-2010 (Alternate Healthy Eating Index 2010) Scoring: 0–110 (higher score = healthier)	Vegan athletes: 78.24 Omnivorous athletes: 68.27
Doumtsios et al. (2010) [44]	Europe (Greece)	Male sailboarders (2.9 ± 1.0 h training/day)	≥18	91	3-day weighed food record	HEI-1999–2000 (Health Eating Index 1999–2000) Scoring: <51 = poor 51–80 = needs improvement >80 = good	WF (Wave and freestyle): 54.3 SF (Slalom and formula): 58.1 **
Gacek et al. (2022) [30]	Europe (Poland)	Male polish elite team athletes (basketball, volleyball, football and handball)	18–38	213	beliefs and eating habits questionnaire (Kom-PAN)	pHDI-10 (Pro-Healthy Diet Index) and nHDI-14 (Non-Healthy Diet Index) Scoring (for both indices): 0–33 = Low 34–66 = Medium 67–100 = High	pHDI-10 score: 19.16 ± 5.80 nHDI-14 score: 15.69 ± 5.67
Gieng et al. (2023) [31]	USA	Female NCAA Division I athletes (soccer, swimming, basketball, cross country and gymnastics)	18–21	41	three interview-based multiple-pass 24 h recall including supplements	HEI-2015 (Healthy Eating Index 2015) Scoring: <51 = poor 51–80 = needs improvement >80 = good	All athletes: 56.2 ± 13
Green et al. (2020) [37]	USA	Female NCAA Division I gymnasts	18–22	11	5-day food record (ASA24-2016 (Automated Self-Administered 24 h Dietary Assessment Tool 2016))	HEI-2015 Scoring: <51 = poor 51–80 = needs improvement >80 = good	Aug: 60.45 ± 11.08 (baseline) Dec: 60.10 ± 12.03 (post-preseason) Apr: 61.08 ± 8.45 (post-competition)
Haubenstricker et al. (2023) [42]	USA	Female competitive bodybuilders (In-season and off-season)	≥18	277	24hR (24-h Recall) (ASA24 2020 (Automated Self-Administered 24 h Dietary Assessment Tool 2020))	HEI-2015 Scoring: <51 = poor 51–80 = needs improvement >80 = good	In season: 70.2 Off season: 68.2 Total: 69.2
Joaquim et al. (2019) [40]	South America	Male and female Brazilian paralympic track-and-field team sprinters	18–38	20	Photographic register (four consecutive days)	HEI-R (Revised Healthy Eating Index) Scoring: <51 = poor 51–80 = needs improvement >80 = good	Males: 61.3 ± 5.3 Females: 63.7 ± 5.9
Jontony et al. (2020) [36]	USA	Female rowing, swimming and gymnastics athletes; Male swimming and wrestling team athletes	≥18	129	VioScreen Web-based, self-admin 30-day graphical FFQ (Food Frequency Questionnaire)	HEI-2015 Scoring: <51 = poor 51–80 = needs improvement >80 = good	All athletes: 71.0 ± 11.2 Female rowing team: 73.5 ± 9.7 Male wrestling team: 56.5 ± 5.7
Kosendiak et al. (2023) [45]	Europe (Poland)	Male and female amateur ultramarathon runners	18–65	308	KomPAN^®^ Dietary Habits and Nutrition Beliefs Questionnaire	HDI-10 & UDI-14 (Unhealthy Diet Index) Scoring: HDI-10: Low = 0–6.66 Moderate = 6.67–13.33 High = 13.34–20 UDI-14: Low = 0–9.33 Moderate = 9.34–18.66 High = 18.67–28	HDI-10 Score: Male (median): 25.00 Female (median): 29.60 UDI-14 Score: Male (median): 13.07 Female (median): 8.93
Lawson et al. (2020) [38]	USA	Male NCAA Division I collegiate football players	18–24	55	ASA24 2019	HEI-2015 Scoring: <51 = poor 51–80 = needs improvement >80 = good	Baseline: 47.7 ± 11.9 Week 8: 51.3 ± 12.3
Murphy & O’Reilly (2021) [46]	Europe (Ireland)	Male hurling athletes (elite and sub elite)	≥18	265	ARFS (Australian Recommended Food Score (ARFS)) (Validated FFQ)	ARFS Scoring: <33 Needs work 33–38 Getting there 39–46 Excellent 47+ Outstanding	Elite (median): 35 Sub-elite (median): 32
Ratajczak et al. (2021) [47]	Europe (Poland)	Male masters athletes (Polish, French and British)	36–65	86	Dietary Habits and Nutrition Beliefs Questionnaire (KomPAN^®^)	pHDI-10 Scoring: 0–33 = Low 34–66 = Medium 67–100 = High	Polish athletes (median): 25.50 French athletes (median): 29.75 British athletes (median): 31.00
Schneider et al. (2023) [41]	South America (Brazil)	Male and female paralympic athletes from 13 different (unspecified) sports	≥18	101	two or four non-consecutive 24hRs	BHEI-R (Brazilian Healthy Eating Index-Revised) and GDQS (Global Diet Quality Score) Scoring for BHEI-R: <51 points (poor diet) 51–80 (needing modification) >80 (healthy diet) Scoring for GDQS: <15 (high risk of NCD (Noncommunicable disease)) 15–23 (moderate risk) >23 (low risk)	BHEI-R score: 60.1 ± 9.5 GDQS score: 18.7 ± 3.8
Skinner et al. (2022) [39]	USA	Female athletes (registered in sports at NCAA DI, National Junior College Athletic Association or the Student Club level)	≥18	120	-	REAP (Rapid Eating Assessment for Patients Questionnaire) Scoring: ^a^ 27–75 (higher score = healthier)	REAP score (median): 53
Taheri et al. (2023) [32]	Trans-national	Male and female elite/sub-elite and recreational athletes (ball sports, athletics, gymnastics and strength, swimming, combat sports and martial arts, rowing and kayaking, cycling, others)	≥18	1420	-	REAP-S (Rapid Eating and Activity Assessment for Patients Short Version) Scoring: 13–39 (higher score = healthier) (Johnston et al., 2018 [49])	Elite athletes: 23.69 ± 7.44 Sub-Elite athletes: 24.69 ± 7.48 All athletes: 24.29 ± 7.48
Tsoufi et al. (2017) [33]	Europe (Greece)	Male elite basketball players	24 ± 4	15 (one team)	24hR through dietitian-run interviews (two training and two competition days per person)	HEI-2005 (Healthy Eating Index 2005) Scoring: <60 = Low 60–79.99 = Average >80 = Adequate	Training Days (median): 89.7 Competition Days (median): 92.7
Turner-McGrievy et al. (2016) [43]	USA	Male and female ultramarathon and other long-distance runners	≥18	422	-	REAP Scoring: ^a^ 13–52 (higher score = healthier) (Gans et al., 2006) [50]	Ultramarathon runners: 33.9 ± 4.9 Half & full marathon runners: 33.3 ± 4.8
Werner et al. (2022) [35]	USA	Male and female NCAA Division I college athletes (football, ice hockey, cross country, golf, soccer, swim and dive, track and field, baseball, tennis, wrestling, basketball, rowing, field hockey, and gymnastics)	≥18	94	ASA24 2018	HEI-2015 Scoring: <51 = poor 51–80 = needs improvement >80 = good	All athletes: 59.2 ± 16.6

* All findings are reported in means unless otherwise stated. ** Wave and freestyle/slalom and formula are two different types of sailboarding that vary in objective and consequently muscular recruitment [44]. ^a^ Same DQI used, but different scoring applied.

**Table 2:** Summary of diet quality as measured by DQI’s in included studies.

Author	Athlete Type	Diet Quality
Craddock et al. [48]	Male endurance athletes	Adequate ^
Doumtsios et al. [44]	Male sailboarders	Needs Improvement
Gacek et al. [30]	Male polish elite mixed team athletes	Poor
Gieng et al. [31]	Female NCAA Division I mixed athletes	Needs Improvement
Green et al. [37]	Female NCAA Division I gymnasts	Needs Improvement
Haubenstricker et al. [42]	Female competitive bodybuilders	Needs Improvement
Joaquim et al. [40]	Male and female Brazilian paralympic track-and-field athletes	Needs Improvement
Jontony et al. [36]	Mixed male and female athletes	Needs Improvement
Kosendiak et al. [45]	Male and female amateur ultramarathon runners	Needs Improvement
Lawson et al. [38]	Male Division I collegiate football players	Poor
Murphy and O’Reilly [46]	Male hurling athletes	Needs Improvement
Ratajczak et al. [47]	Male Polish, French, and British masters athletes	Poor
Schneider et al. [41]	Male and female mixed paralympic athletes	Needs Improvement
Skinner et al. [39]	Female mixed athletes	Adequate ^
Taheri et al. [32]	Male and female recreational to elite mixed sport athletes	Needs Improvement ^
Tsoufi et al. [33]	Male elite basketball players	Adequate
Turner-McGrievy et al. [43]	Male and female long-distance runners	Needs Improvement ^
Werner et al. [35]	Male and female NCAA Division I mixed sport athletes	Needs Improvement

^ These studies used a diet quality index comprised of a continuous numerical scoring scale and did not have a categorical matrix, i.e., “Poor”, “Needs Improvement”, and “Adequate”. In these instances, the average diet quality score of athletes in these studies was divided by the total available points from the DQI tool providing a percentage. The following criteria were then applied to provide a generalised diet quality assessment: 0–33% = Poor, 34–66% = Needs Improvement, 67–100% = Adequate. This scoring aligned with the percentages used in the Healthy Diet Index-10 (HDI-10) and Unhealthy Diet Index-14 (UDI-14) [45].

## Supplementary Materials

The following supporting information can be downloaded at: https://www.mdpi.com/article/10.3390/nu17010089/s1, Table S1: Summary of studies evaluating diet quality in athletes (not peer reviewed). References [75,76,77,78,79] are cited in the supplementary materials.
