# Supplementary material for: The Diet Quality of Athletes as Measured by Diet Quality Indices: A Scoping Review
Source: Nutrients. 2024 Dec 29;17(1):89. doi: 10.3390/nu17010089 (PMC11722857; doi:10.3390/nu17010089)
Supplement: Supplementary file 1 [file nutrients-17-00089-s001.zip › nutrients-3390790-supplementary.pdf]

Supplementary Material

Table S1. Summary of studies evaluating diet quality in athletes (not peer reviewed).

Author/s (Year)	Country	Athlete Type (Gender)	Age	n	Dietary Assessment Method	Tool Diet Quality Index (DQI)	Findings*
Hemme (2023)[1]	USA	Injured/recently injured male and female collegiate athletes from multiple NCAA Division III athletic departments	18-25yo	48	-	REAP-S<br><br>Scoring:<br>13-39 (higher score = healthier)<br>(Johnston et al. 2018)[2]	REAP-S score: 28.98±3.5
Renaldo (2022)[3]	USA	Male and female student athletes of various collegiate sports	18-30yo	33 college students (athletes [n=19] + physically active students)	-	REAP-S<br><br>Scoring:<br>13-39 (higher score = healthier)<br>(Johnston et al., 2018)[2]	REAPS score:<br>Student athletes: <32
Salleh & Samri (2022)[4]	Malaysia	Male and female collegiate athletes (various sports)	18-30yo	195	DQI-I questionnaire	DQI-I-2003[1]<br><br>Scoring:<br>0-100 (higher score = healthier)	DQI-I scores:<br>Male: 60.4±13.9<br>Female: 63.1±12.6<br>All athletes: 61.8±13.2
Skinner (2020)[5]	USA	NCAA Female Athletes (including sports such as soccer (17%), softball (12%), or track and field (20%))	≥18yo	120	-	REAP<br><br>Scoring:<br>27-75 (higher score = healthier)	REAP score for all athletes (median):<br>57
Webber et al. (2015)[6]	USA	Male and female athletes from various collegiate sports (gymnastics, swimming, diving, soccer, basketball, volleyball)	≥18 (average 19.4yo)	138	Block FFQ (2005)	HEI-2005<br><br>Scoring:<br><60 = Low<br>60 – 79.99 = Average<br>>80 = Adequate	HEI-2005 scores:<br>Males: 47.7±7.9<br>Females: 51.3±8.6

[1] Diet Quality Index International 2003

Werner (2021)[7]	USA	Male & female collegiate athletes (Football, Basketball, Ice Hockey, Cross Country, Golf, Soccer, Swim and Dive, Track and Field, Baseball, Rowing, Tennis, Field Hockey, Wrestling, Gymnastics)	≥18yo	94	ASA24 (24hR)	HEI-2015<br><br><u>Scoring</u>:<br><51 = poor<br>51-80 = needs improvement<br>>80 = good	<u>HEI-2015 scores</u>:<br>All athletes: 59.2±16.6

[*] All findings are reported in means unless otherwise stated.

1. Hemme, T.G., *Diet Quality and the Psychological Response to Sport Injury*. 2023.
2. Johnston, C.S.; Bliss, C.; Knurick, J.R., and Scholtz, C., Rapid Eating Assessment for Participants [shortened version] scores are associated with Healthy Eating Index-2010 scores and other indices of diet quality in healthy adult omnivores and vegetarians. *Nutr J*, 2018. 17(1): p. 89.
3. Renaldo, M., *Burnout, Diet Quality, and Sleep Quality in College Student Athletes vs. Physically Active College Students*. 2022.
4. Salleh, R.M. and Samri, A.N., *Influence of Food Purchase Behaviour on Diet Quality of University Athletes during COVID-19 Lockdown*. 2022. p. 501-506.
5. Skinner, J., *Relationship of Nutrition Knowledge and Dietary Intake Among Collegiate Female Athletes*. 2020.
6. Webber, K.; Stoess, A.I.; Forsythe, H.; Kurzynske, J.; Vaught, J.A., and Adams, B., Diet quality of collegiate athletes. *College Student Journal*, 2015. 49(2): p. 251-256.
7. Werner, E.N., *Sport Nutrition Knowledge and Dietary Habits in College Athletes*. 2021.
